# A Mystery

**Published:** 1890-02

**Authors:** 


### A MYSTERY.

Daisy Robinson, a little colored girl of Sumter, Canada, about twelve years of age, is an ordinary looking child, and has a languid look in her eyes. She seldom smiles, and gazes listlessly around at the crowds of people who are continually going to see her. Daisy's mother is dead, and she lives with an aunt.

As an illustration of what dozens of eminently respectable and trustworthy people of Sumter testify to, Policeman Epperson, who was detailed to investigate the matter, makes the following statement: "I went into the room where the girl and her sister were, and turned everybody else out, and sat on the bed facing the fireplace with my lantern in my hand. There was another light in the room and a fire in the fireplace. The girl sat on the right of the chimney, facing me, her chair leaning back against the chimney, her hands in her lap.

"I waited awhile and nothing occurring, I was about to leave, when a dinner plate that was on the top of the bureau to my left and against the wall, several feet away from the girl, came sailing out into the air and fell right side up on the floor, breaking into pieces. A two-quart bucket on the mantel-shelf then sprang out and fell to the floor at my feet. Immediately after a trivet, weighing five or six pounds, that stood by the fire, dashed out and across to where I was, striking on one of its feet and spinning around until it came to a rest.

"Then a shovel which was back in the corner to the right of the girl, came prancing out and fell with a clatter. All this time, as it seemed to me, the girl was sitting perfectly quiet and there was no way for the things to be thrown in from the outside. Besides, I saw the missiles as they were coming through the air, and they came from just where they had been lying all the while and I am satisfied that no one in or out of the room moved or threw them."

The room was afterwards examined and found to be closely sealed above and all round. The floor is tight and a careful examination from the outside failed to reveal a crack that even a ten-cent piece could have been thrown through.

Similar occurrences manifest themselves in every house or room where Daisy goes, so that she is not a desirable visitor. Her aunt says she cannot afford such costly entertainment for the public and does not know what to do in the matter, as she cannot turn her out of doors. She says that this morning, while Daisy was eating her breakfast, the bedstead in her room was actually wrenched to pieces by an unseen power.—Extract from *Truth*, Toronto.

